# A quasi-experimental study estimating the impact of long-lasting insecticidal nets with and without piperonyl butoxide on pregnancy outcomes

**DOI:** 10.1186/s12936-021-04034-0

**Published:** 2022-01-04

**Authors:** Michelle E. Roh, Brenda Oundo, Grant Dorsey, Stephen Shiboski, Roly Gosling, M. Maria Glymour, Sarah G. Staedke, Adam Bennett, Hugh Sturrock, Arthur Mpimbaza

**Affiliations:** 1 Malaria Elimination Initiative, Institute of Global Health Sciences, University of California, San Francisco, CA USA; 2 Infectious Diseases Research Collaboration, Kampala, Uganda; 3 Division of HIV, Infectious Diseases, and Global Medicine, Department of Medicine, University of California, San Francisco, CA USA; 4 Department of Epidemiology and Biostatistics, University of California, San Francisco, CA USA; 5 London School of Hygiene & Tropical Medicine, London, UK; 6 Child Health and Development Centre, College of Health Sciences, Makerere University, Kampala, Uganda

**Keywords:** Plasmodium falciparum, Malaria in pregnancy, Long-lasting insecticidal net, Low birthweight, Stillbirth, Difference-in-differences, Interrupted time series, Piperonyl butoxide, Pyrethroid resistance

## Abstract

**Background:**

Long-lasting insecticidal nets (LLINs) are the main vector control tool for pregnant women, but their efficacy may be compromised, in part, due to pyrethroid resistance. In 2017, the Ugandan Ministry of Health embedded a cluster randomized controlled trial into the national LLIN campaign, where a random subset of health subdistricts (HSDs) received LLINs treated with piperonyl butoxide (PBO), a chemical synergist known to partially restore pyrethroid sensitivity. Using data from a small, non-randomly selected subset of HSDs, this secondary analysis used quasi-experimental methods to quantify the overall impact of the LLIN campaign on pregnancy outcomes. In an exploratory analysis, differences between PBO and conventional (non-PBO) LLINs on pregnancy outcomes were assessed.

**Methods:**

Birth registry data (n = 39,085) were retrospectively collected from 21 health facilities across 12 HSDs, 29 months before and 9 months after the LLIN campaign (from 2015 to 2018). Of the 12 HSDs, six received conventional LLINs, five received PBO LLINs, and one received a mix of conventional and PBO LLINs. Interrupted time-series analyses (ITSAs) were used to estimate changes in monthly incidence of stillbirth and low birthweight (LBW; <2500 g) before-and-after the campaign. Poisson regression with robust standard errors modeled campaign effects, adjusting for health facility-level differences, seasonal variation, and time-varying maternal characteristics. Comparisons between PBO and conventional LLINs were estimated using difference-in-differences estimators.

**Results:**

ITSAs estimated the campaign was associated with a 26% [95% CI: 7–41] reduction in stillbirth incidence (incidence rate ratio (IRR) = 0.74 [0.59–0.93]) and a 15% [-7, 33] reduction in LBW incidence (IRR=0.85 [0.67–1.07]) over a 9-month period. The effect on stillbirth incidence was greatest for women delivering 7–9 months after the campaign (IRR=0.60 [0.41–0.87]) for whom the LLINs would have covered most of their pregnancy. The IRRs estimated from difference-in-differences analyses comparing PBO to conventional LLINs was 0.78 [95% CI: 0.52, 1.16] for stillbirth incidence and 1.15 [95% CI: 0.87, 1.52] for LBW incidence.

**Conclusions:**

In this region of Uganda, where pyrethroid resistance is high, this study found that a mass LLIN campaign was associated with reduced stillbirth incidence. Effects of the campaign were greatest for women who would have received LLINs early in pregnancy, suggesting malaria protection early in pregnancy can have important benefits that are not necessarily realized through antenatal malaria services. Results from the exploratory analyses comparing PBO and conventional LLINs on pregnancy outcomes were inconclusive, largely due to the wide confidence intervals that crossed the null. Thus, future studies with larger sample sizes are needed.

## Background

In sub-Saharan Africa, malaria in pregnancy is a major public health problem. In 2019, an estimated 12 million pregnant women were exposed to the *Plasmodium* parasite [1]. Infection with malaria parasites during pregnancy is known to increase the risk of low birthweight and stillbirth delivery [2]. Prevention and prompt case management are key strategies for reducing the adverse effects of malaria in pregnancy. In areas of moderate-to-high malaria transmission, the World Health Organization (WHO) recommends a package of interventions including the use of long-lasting insecticidal nets (LLINs), intermittent preventive treatment of malaria in pregnancy, and prompt management of clinical cases [3].

LLINs have played a crucial role in malaria control. Between 2004 and 2019, nearly 2.9 billion LLINs were distributed to malaria endemic areas [1, 4]. Currently, pyrethroids are the only WHO-certified class of insecticide currently recommended for use in LLINs and there is increasing concern that the spread of pyrethroid-resistant mosquitoes may reduce the efficacy of LLINs for malaria prevention. Though the evidence base is inconsistent on the extent to which pyrethroid resistance affects LLIN efficacy [5–7], in 2019, 73 malaria-endemic countries reported some level of pyrethroid resistance [1], which has prompted the urgent search for alternative LLINs that can overcome or slow its spread.

Premised on these concerns, several agencies have been actively working to develop new LLINs. For these LLINs to be more effective, they must overcome one of two mechanisms associated with pyrethroid resistance: (1) knockdown resistance (*kdr*) caused by single-point mutations in the voltage-gated sodium channel where pyrethroids bind and (2) metabolic resistance through mutations in *cytochrome P450 (CYP450)* genes [8–10]. In 2017, the WHO released a conditional statement endorsing a new type of pyrethroid-based LLIN treated with piperonyl butoxide (PBO), a chemical synergist known to inhibit CYP450 enzyme activity [11]. The recommendation, based on promising results from a Tanzanian trial [12], called for the deployment of PBO LLINs in areas where pyrethroid resistance is driven partly by metabolic-based mechanisms [11, 13].

Shortly after the WHO recommendation, the Ugandan National Malaria Control Programme (NMCP) conducted a national LLIN distribution campaign. Nested within the campaign was a cluster randomized controlled trial which compared the effectiveness of PBO LLINs to conventional (non-PBO) LLINs on parasite prevalence [14]. Six months after LLINs were distributed, parasite prevalence in children 2–10 years of age was 26% [95% CI: 13, 28] lower in the PBO LLIN group (11%) compared to conventional LLIN group (15%), after controlling for baseline differences. However, reductions from baseline were seen in both groups, suggesting conventional LLINs may still provide a protective effect.

The objectives of this study were to assess whether the national LLIN campaign was associated with improved pregnancy outcomes and whether PBO LLINs conferred a greater protective effect than conventional LLINs.

## Methods

### Study setting

Between March 2017 and March 2018, the Uganda NMCP and research collaborators conducted the LLINEUP trial [14], a large-scale cluster randomized controlled trial which randomized 104 health subdistricts (HSDs) in the Eastern (n = 38) and Western (n = 66) regions of Uganda to receive PBO LLINs (PermaNet 3.0 or Olyset Plus) or conventional LLINs (PermaNet 2.0 or Olyset Net). In this trial, not all clusters received the allocated LLINs: three HSDs received a mixture of PBO and conventional LLINs.

**Fig. 1:**
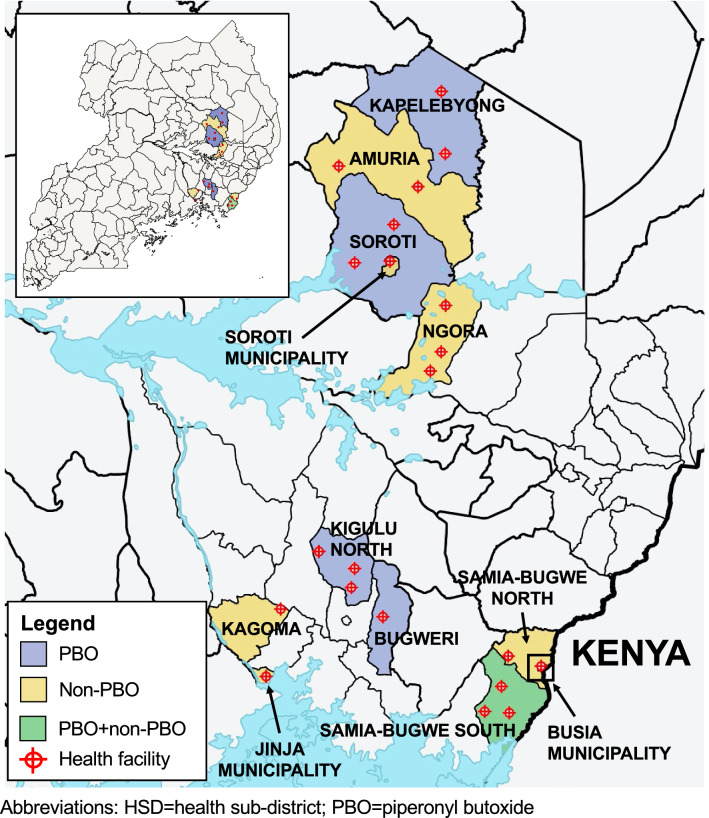
Map of the study health sub-districts (HSDs) (n = 12) and health facilities (n = 21). Purple shaded areas indicate HSDs that received PBO long-lasting insecticidal nets (LLINs); yellow shaded areas indicate HSDs that received conventional (non-PBO) LLINs; and the green shaded area indicates the HSD that received a mix of PBO and conventional LLINs. Red points indicate the location of study health facilities where delivery information was collected

This secondary analysis study selected a subset of HSDs from the Eastern Region (12/38; 32%) (Fig. 1) to evaluate the overall impact of the LLIN campaign on stillbirth and low birthweight (LBW) incidence and to assess whether there were differences in the impact between PBO and conventional LLINs. Study sites were non-randomly selected based on the availability of health facility data within these HSDs. Of the 12 HSDs selected for this study, six received conventional LLINs (Amuria, Jinja Municipality, Kagoma, Ngora, Samia-Bugwe North, and Soroti Municipality), five received PBO LLINs (Bugweri, Busia Municipality, Kapelebyong, Kigulu North, and Soroti), and one HSD (Samia-Bugwe South) received a mix of PBO and conventional LLINs. Data from Samia-Bugwe South was used to assess the overall impact of the mass LLIN campaign but excluded from analyses comparing PBO to conventional LLINs. Timing of the LLIN campaign varied across HSDs, where five HSDs received LLINs in late March 2017 and seven HSDs received LLINs in mid-May 2017. Baseline entomological survey data from the LLINEUP trial [14] indicated *Anopheles gambiae sensu lato* (s.l*.*) and *Anopheles funestus* (s.l.) mosquitoes exhibited moderate-to-high allele frequencies of both the kdr- and metabolic-based mutations associated with pyrethroid resistance. In the eastern region of Uganda, the allele frequencies of *Vgsc*-L1014S and *Vgsc*-L1014 mutations were 1.00 and 0.06, respectively, and the allele frequency of the *Cyp4j5*-L43F mutation ranged from 0.60 to 0.80 [8]. Prior studies conducted in Eastern Uganda demonstrated an *An. gambiae* mortality ranging from 25 to 87% upon exposure to pyrethroid class insecticides, indicative of a high level of pyrethroid resistance [15, 16].

## Data source

**Fig. 2:**
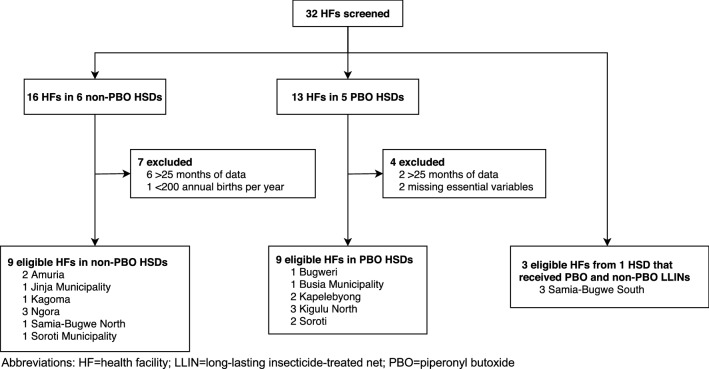
Flowchart of health facility selection

To assess birth outcome trends before and after the LLIN campaign, individual-level birth records were obtained from hard copies of the Integrated Maternity Registry, captured through the Health Management Information System (HMIS), a routine surveillance system designed to monitor disease and health trends by the Ministry of Health [17]. This registry, managed by trained nurses and midwives, includes data on delivery outcomes (e.g., date of delivery, birthweight, and stillbirth) and maternal characteristics (e.g., age, gravidity, and HIV status). From eligible health facilities (described below), individual birth records were collected approximately 29 months before and 9 months after LLIN distribution (January 2015-February 2018). Health facilities from each HSD were considered eligible if they were: (1) government-operated; (2) included a maternity ward; (3) located >5 km from a neighbouring HSD (to mitigate bias from exposure misclassification); and (4) had a mean delivery rate of >200 deliveries per year. Due to concerns over data quality [18, 19], health facilities were screened by a study coordinator and excluded if: (1) data were missing for >25 months during the study period or (2) covariates or outcomes were systematically missing. Of the 32 screened health facilities, eight were excluded due to missing >25 months of data, two were excluded due to systematic missingness of the outcome or covariate data, and one was later found to have a mean delivery rate of <200 deliveries per year. The final analytic sample included data from 39,085 deliveries across 21 health facilities (Fig. 2). Analyses were conducted at the health facility-level by aggregating data to the health facility and month.

### Measurements

#### Treatment variable

To assess the impact of distribution of any LLINs, the intervention period was defined as the cumulative 9-month post-LLIN period. To assess for dose-dependent effects, the post-LLIN campaign period was further categorized into 3-month intervals; months 1–3, 4-6, and 7–9 to approximate the third, second, and first pregnancy trimesters, respectively. To compare between net types, treated and comparison units were defined as HSDs that received PBO and conventional LLINs, respectively.

#### Outcomes

The outcomes evaluated in this study were incidence of stillbirth and low birthweight (LBW; defined as birthweight <2,500 g among live births) assessed among singleton deliveries.

### Statistical analysis plan

#### Impact of the LLIN campaign (irrespective of net type)

Interrupted time series analyses (ITSA) using segmented regression [20–23] were used to quantify the impact of the overall LLIN campaign. To estimate the counterfactual number of LBW and stillbirth deliveries that would have occurred in absence of the LLIN campaign, ITSA assumes the pre-intervention trend would have continued had it not been ‘interrupted’ by the LLIN campaign [21]. To estimate the impact of the campaign, observed and unobserved counterfactual outcomes (which was estimated by extrapolating the pre-intervention trend) were compared during the post-intervention period.

ITSA were conducted using Poisson regression to estimate the monthly number of LBW and stillbirth deliveries using data aggregated to the health facility-month. Models included the following covariates: a linear term for months since the start of the study (to capture the pre-LLIN trend); a linear term for months after the LLIN campaign (to capture the post-LLIN trend); calendar month fixed effects (to account for seasonality); health facility fixed effects (to account for group-level variation); and time-varying maternal characteristics (i.e., mean maternal age at delivery, proportion of primigravidae, and proportion of HIV-positive women). The log of the number of deliveries per health facility-month was included as an offset term and robust standard errors were used to account for autocorrelated errors. Models were then used to predict the unobserved counterfactual outcomes (i.e., the “expected” outcomes during the post-LLIN period in absence of the LLIN campaign) for each health facility in each month. Incidence rate ratios (IRRs) were calculated by dividing the sum of the observed outcomes by the estimated counterfactual outcomes for each of the pre-specified post-LLIN campaign periods, assuming that the denominator (at-risk population) would have been the same for observed and unobserved counterfactual groups. 95% confidence intervals were generated using a block-bootstrapping procedure to account for clustered observations at the health facility-level.

#### Comparison of PBO and conventional LLINs

Difference-in-differences analyses [24] were used to determine whether PBO LLINs conferred a greater protective effect than conventional LLINs. Unlike ITSA, which uses the pre-intervention trend to estimate counterfactual outcomes, difference-in-differences uses pre-post observations from a contemporaneous control group (conventional LLIN) to estimate the unobserved counterfactual trend. Poisson regression was used to estimate the monthly number of LBW and stillbirth deliveries per health facility-month. Models included: the “treatment” variable (an indicator variable for the post-LLIN period for PBO HSDs); health facility fixed effects (to control for group-level differences), monthly fixed effects to control for time-varying, but group-invariant differences (e.g., changes in IPTp scale-up over time), and the time-varying maternal characteristics included in ITSA models. The HSD that received a mix of both LLIN types was excluded from difference-in-differences analyses.

Valid causal inference from difference-in-differences analyses relies on the assumption that PBO and conventional LLIN groups would have shared parallel trends had the PBO LLIN group received conventional LLINs [24]. Though the validity of this assumption cannot be proven, pre-LLIN trends were tested to assess whether trends were parallel between PBO and conventional LLIN groups using an interaction term between a binary indicator of the PBO LLIN group and a linear time trend. Testing of parallel trends were conducted using data from the pre-campaign period. Pre-LLIN trends appeared to be similar between PBO and conventional LLIN groups for LBW (p = 0.75), but not stillbirth models (p = 0.083). Thus, group-specific linear time trends were included in stillbirth difference-in-differences models using health facility*time interaction terms. This alternative difference-in-difference specification relaxes the parallel trends assumption by allowing pre-intervention trends to vary by group, but assumes that in absence of the campaign, the rate of change between PBO and conventional LLIN groups would have had parallel [25, 26]. Analyses were conducted in R (version 3.5.3) and Stata (StataCorp LLC, version 16.1).

## Results

### Descriptive analysis

Over the 38 months of observation (January 2015 to February 2018), data on 39,085 singleton deliveries were available from five HSDs that received conventional LLIN (n = 13,156), six HSDs that received PBO LLINs (n = 18,353), and one HSD that received a mixture of conventional and PBO LLINs (n = 7576). Approximately 3.3% of deliveries were stillbirths (n = 1279) and of the total live births (n = 37,806), 4.6% (n = 1727) were LBW.

Table 1 presents the characteristics and delivery outcomes of the study population for each LLIN group during the pre- and post-campaign periods. Overall, monthly averages of maternal age at delivery and HIV prevalence within health facilities were similar across LLIN groups and pre- and post-LLIN campaign periods. The proportion of primigravidae was generally higher during post-campaign months, but this finding was consistent between LLIN groups. The mean number of monthly deliveries across health facilities was 57.6 (standard deviation (SD): 36.6). The mean monthly LBW delivery rate was similar across LLIN groups but was generally lower during the post-LLIN campaign period. The mean monthly stillbirth delivery rate differed across LLIN groups but was lower during post-LLIN campaign months for the PBO group and conventional LLIN group. Samia-Bugwe South, the HSD that received a mixture of PBO and conventional LLINs, had a higher mean monthly stillbirth delivery rate compared to PBO and conventional LLIN groups, and this rate was higher during post-LLIN campaign months.

**Table 1:** Study population characteristics across LLIN groups stratified by pre- and post-campaign periods. Summary statistics are presented as monthly averages/proportions across health facilities

	Conventional LLINs		PBO LLINs		Conventional + PBO LLINs	
	Pre-period	Post-period	Pre-period	Post-period	Pre-period	Post-period
Total number of observations	9129	4027	13,880	4473	5754	1822
Maternal age in years at delivery, mean (SD)	24.5 (1.2)	24.3 (1.0)	24.5 (1.0)	24.4 (1.9)	23.6 (0.9)	23.6 (0.6)
% Primigravidae, mean (SD)	22.4 (10.5)	25.8 (10.8)	19.4 (10.5)	23.6 (9.4)	29.9 (8.1)	32.1 (4.6)
% HIV prevalence, mean (SD)	3.2 (2.9)	3.2 (3.4)	3.3 (2.9)	3.5 (2.5)	3.5 (2.9)	3.8 (2.5)
Birth outcomes						
Number of deliveries, mean (SD)	44.8 (19.7)	49.7 (25.1)	62.0 (42.6)	66.8 (48.0)	71.0 (36.3)	86.8 (41.3)
LBW infants per 100 births, mean (SD)	4.5 (4.7)	3.9 (4.1)	4.0 (4.2)	4.3 (3.8)	5.3 (4.2)	4.3 (2.6)
Stillbirths per 100 deliveries, mean (SD)	1.7 (7.2)	1.1 (2.2)	3.9 (5.2)	3.3 (4.3)	4.0 (2.3)	5.2 (4.0)

*LBW *low birthweight, *LLIN *long-lasting insecticidal net, *PBO *piperonyl butoxide, *SD *standard deviation

## Impact of LLIN campaign (irrespective of net type)

**Fig. 3:**
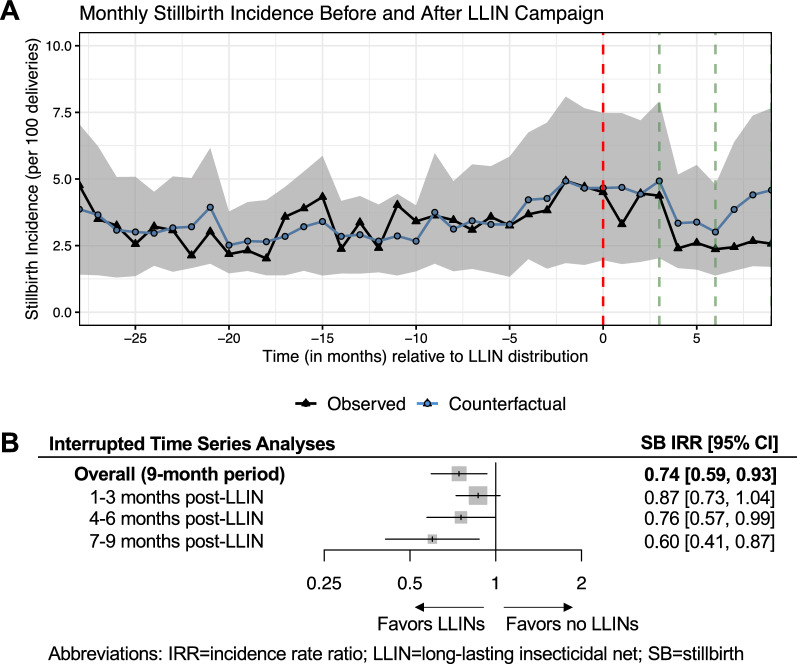
Association between the LLIN campaign and stillbirth incidence estimated from interrupted time series analyses (ITSA). **A** shows the observed and unobserved counterfactual number of stillbirth deliveries per month summed across all health facilities. The red vertical line marks the timepoint when LLINs were distributed, and green vertical lines indicate the three- and six-month cut-off points after LLIN distribution. The grey shaded region represents the 95% confidence intervals estimated from ITSA models using a block-bootstrapping procedure accounting for clustered observations at the health facility-level. **B** shows the effect estimates produced by dividing the sum of the observed number of stillbirths by the unobserved (“expected”) counterfactual number of stillbirths estimated from ITSA models. Overall estimates and those stratified by three-month post-LLIN intervals are presented as incidence rate ratios (IRRs)

Over a nine-month period, the incidence rate ratio (IRR) estimating the effect of the LLIN campaign on stillbirth incidence was IRR = 0.74 [95% CI: 0.59, 0.93] (Fig. 3). The effect of the campaign appeared to be dose-dependent, such that the effects were greater among women for whom the LLIN campaign occurred earlier in their pregnancy (Fig. 3B). Among women delivering 7–9 months after the campaign (i.e., women who might have benefited from the impact of the LLIN campaign throughout pregnancy), the relative reduction in the incidence rate was 40% [95% CI: 13, 59] (IRR = 0.60 [95% CI: 0.41, 0.87]).

**Fig. 4:**
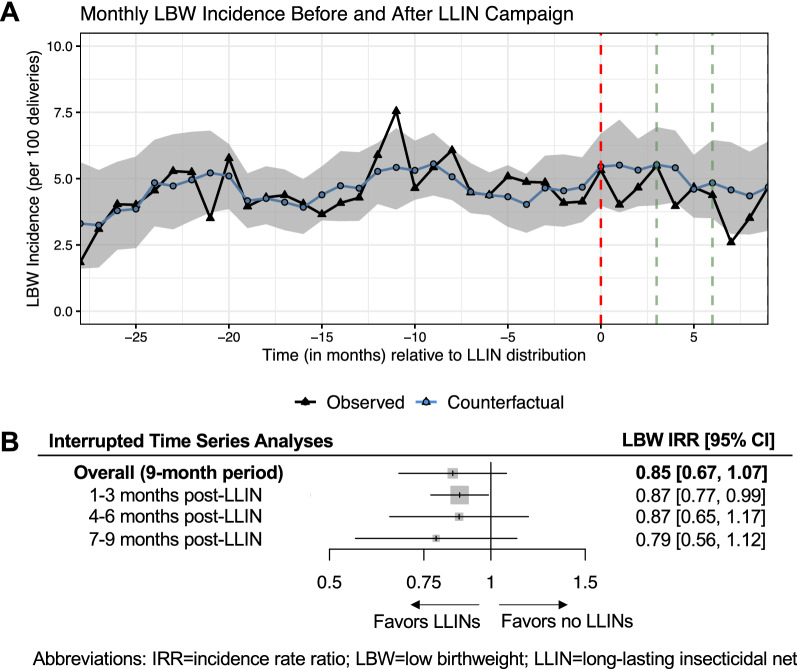
Association between the LLIN campaign and low birthweight (LBW) incidence estimated from interrupted time series analyses (ITSA). **A** shows the observed and unobserved counterfactual number of LBW deliveries per month summed across all health facilities. The red vertical line marks the timepoint when LLINs were distributed, and green vertical lines indicate the three- and six-month cut-off points after LLIN distribution. The grey shaded region represents the 95% confidence intervals estimated from ITSA models using a block-bootstrapping procedure accounting for clustered observations at the health facility-level. **B** shows the effect estimates produced by dividing the sum of the observed number of LBW deliveries by the unobserved (“expected”) counterfactual number of LBW deliveries estimated from ITSA models. Overall estimates and those stratified by three-month post-LLIN intervals are presented as incidence rate ratios (IRRs)

The IRR estimated from ITSA of the effect of the LLIN campaign on low birthweight incidence was 0.85 [95% CI: 0.67, 1.07] (Fig. 4). Estimates remained consistent across each three-month post-campaign interval, however, confidence intervals around all LBW effect estimates were generally wide and included or nearly included the null.

## Comparison of PBO and conventional LLINs

**Fig. 5:**
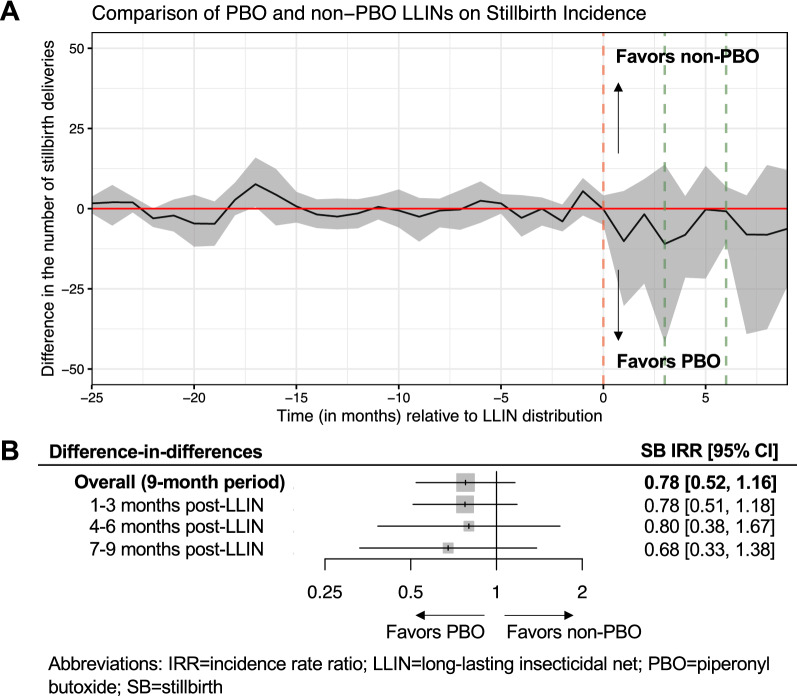
Comparison of PBO and conventional (non-PBO) LLINs on stillbirth incidence estimated from difference-in-differences models. **A** shows month-by-month differences between the observed and unobserved (“expected”) counterfactual number of stillbirth deliveries in the PBO LLIN group had this group received conventional LLINs. The red dotted vertical line marks the timepoint when LLINs were distributed, the green dotted vertical lines indicate the 3- and 6-month cut-off points after LLIN distribution, and the red horizontal solid line is a reference line had there been no difference between PBO and conventional LLINs. The grey shaded region represents the 95% confidence intervals estimated from difference-in-differences estimators using a block-bootstrapping procedure accounting for clustered observations at the health facility-level. **B** shows the difference-in-differences effect estimates stratified by three-month post-LLIN intervals presented as incidence rate ratios (IRRs)

Over the nine-month post-campaign period, the IRR estimated from difference-in-differences analyses comparing the effect of PBO to conventional LLINs on stillbirth incidence was 0.78 [95% CI: 0.52, 1.16] (Fig. 5). The direction of stillbirth estimates appeared to be consistent across each three-month post-campaign interval, however, confidence intervals around all stillbirth IRRs were wide and included the null.

**Fig. 6:**
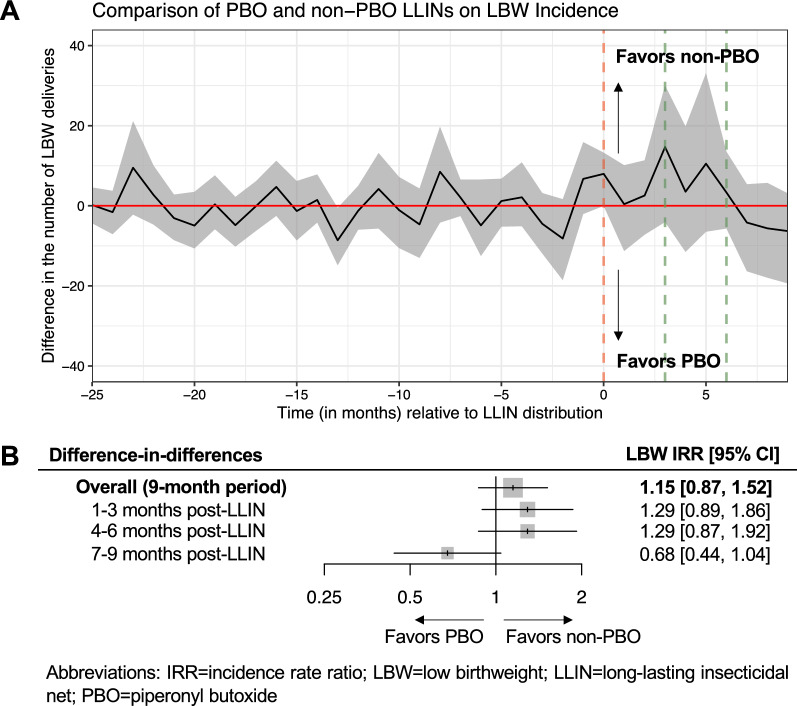
Comparison of PBO and conventional (non-PBO) LLINs on low birthweight (LBW) incidence estimated from difference-in-differences models. Figure A shows month-by-month differences between the observed and unobserved (“expected”) counterfactual number of LBW deliveries in the PBO LLIN group had this group received conventional LLINs. The red vertical dotted line marks the timepoint when LLINs were distributed, the green vertical dotted lines indicate the 3- and 6-month cut-off points after LLIN distribution, and the red horizontal solid line is a reference line had there been no difference between PBO and conventional LLINs. The grey shaded region represents the 95% confidence intervals estimated from difference-in-differences estimators using a block-bootstrapping procedure accounting for clustered observations at the health facility-level. Figure B shows the difference-in-differences effect estimates stratified by three-month post-LLIN intervals presented as incidence rate ratios (IRRs)

Over the nine-month post-campaign period, the IRR estimated from difference-in-difference analyses comparing the effect of PBO to conventional LLINs on LBW incidence was 1.15 [95% CI: 0.87, 1.52]. The direction of the effect differed for women delivering 7-9 months after the LLIN campaign (IRR=0.68 [95% CI: 0.44, 1.04]), however, confidence intervals around all LBW estimates were wide and crossed the null (Fig. 6).

## Discussion

In 2017, the Ugandan Ministry of Health and research partners implemented a nationwide LLIN distribution campaign. Embedded within the campaign was a cluster randomized controlled trial comparing PBO to conventional (non-PBO) LLINs on parasite prevalence [14]. Using routine birth surveillance data from a subset of eastern study sites, this goal of this study was to estimate the impact of the campaign on the incidence of birth outcomes. Using interrupted time series analyses, this study found that the LLIN campaign was associated with a 26% reduction in stillbirth incidence over a nine-month period. The impact of the campaign on stillbirth incidence appeared to be dose-dependent, such that women who were likely in their first trimester of pregnancy when the LLIN distribution campaign occurred benefitted more than women who were likely in their second or third trimester. In addition, there was some evidence to suggest that the campaign was associated with reduced LBW incidence, though confidence intervals around this effect estimate crossed the null. In this region of intermediate-to-high levels of metabolic-based mutations associated with pyrethroid resistance, findings were inconclusive on whether PBO LLINs were more beneficial than conventional LLINs on improving pregnancy outcomes, which may have largely been due to the limited sample size.

Consistent with prior studies [27, 28] and WHO guidelines [3], findings from the ITSAs suggest malaria prevention early in pregnancy is critical for improving pregnancy outcomes. However, through the current targetted LLIN delivery approach, women are distributed LLINs at their first antenatal care visit, which often misses the first trimester of pregnancy [29]. This study suggests that LLIN delivery through mass campaigns can help to support existing antenatal care delivery channels by reaching women earlier in pregnancy and to improve overall LLIN coverage among pregnant women, which in sub-Saharan Africa is estimated at 52% (well below the 80% target level) [1]. Furthermore, mass campaigns are likely to increase coverage in the overall population, where pregnant women can benefit from the “community” effects of mass campaigns through reducing malaria transmission intensity.

Exploratory analyses comparing PBO to conventional LLINs showed that relative to conventional LLINs, PBO LLINs may confer greater protection against stillbirths, however, confidence intervals around these effect estimates were wide and crossed the null. While this may have been due to the small sample size (6 PBO versus 5 conventional HSDs), there may be other reasons that could partly explain these findings. First, it is possible that non-random selection of HSDs may have introduced bias that was not fully controlled for in the difference-in-differences analyses. Second, the nine-month follow-up period may have been too short and benefits would have only been seen over a longer period when the community effects of LLINs have had a chance to become established. Third, it is possible that the pyrethroid resistance may not impose a major threat on LLIN effectiveness, as found in cohort studies from Benin, Cameroon, India, Kenya, and Sudan [5]. In the original LLINEUP trial [14], a modest difference in parasite prevalence was observed between PBO and conventional LLINs and reductions from baseline were observed in both PBO and conventional LLIN groups. This suggests that conventional LLINs may still confer some benefit and that perhaps the comparative effect of PBO to conventional LLINs on malaria prevention may not have been substantial enough to translate into significant differences in pregnancy outcomes. Thus, while future studies with larger sample sizes are needed to validate the study findings, combined results from the ITSA and difference-in-differences analyses suggest that conventional LLINs may still reduce adverse pregnancy outcomes.

Another important finding from this study was demonstration of the use of routine surveillance data for conducting impact evaluations of large-scale malaria control interventions on pregnancy outcomes. Traditionally, the impact of mass campaigns of malaria control interventions on pregnant women have relied almost exclusively on measures of coverage and utilization [30] rather than clinically relevant health outcomes. Though these metrics are important, measuring clinically relevant health outcomes can provide national malaria control programmes with a better quantitative assessment of gains achieved through investment in their malaria control strategies [31]. As collecting data on these outcomes may be more time and resource intensive than other more commonly measured outcomes of impact evaluations (e.g., coverage [32], malaria burden reduction [31, 33, 34]), studies should consider using HMIS registries as an alternative data source, as these data encompass a wide range of health outcomes with comprehensive information collected routinely over time. Use of these data to conduct additional impact evaluations may provide better opportunities for national malaria and maternal and child health programs to monitor the effectiveness of interventions on birth outcomes, which, in turn, may guide future local and national policies. However, screening of these HMIS registries will be necessary prior to their use, given concerns regarding data quality and missingness [35].

The study had some limitations. First, selection of study sites was based on data availability and given the small number of HSDs, the study may not have had sufficient statistical power to detect differences between PBO and conventional LLINs, as evidenced by the wide confidence intervals around effect estimates. Second, several health facilities were excluded to ensure the collection of high-quality data, which may have limited the external validity of the findings. Third, though the HMIS database enabled us to capture a comprehensive set of delivery information, variables in this registry may have been measured with error. Measurement error of gravidity and HIV may have reduced the ability to adequately control for these time-varying covariates. Fourth, geographic information on the residence of women delivering at health facilities was not collected due to frequent absence of this variable. Thus, the HSD where the woman gave birth may not accurately represent their HSD of residence. Though, health facilities were selected at least five km away from neighbouring HSDs to reduce this type of non-differential exposure misclassification, effect estimates may have still been biased. Lastly, HSDs were selected non-randomly and though quasi-experimental study designs (i.e., ITSA and difference-in-differences) were used to control for confounding, residual or unmeasured confounding may have biased results. Thus, estimates should be interpreted with caution.

In summary, findings from this study contribute to the current evidence base of the effectiveness of LLINs on pregnancy outcomes and highlight the importance of the first trimester of pregnancy as a critical period for stillbirth and LBW prevention. These results suggest mass distribution campaigns can complement existing antenatal service delivery mechanisms by increasing LLIN use early in pregnancy. Given the lack of clear evidence of the comparative advantage of PBO LLINs to conventional LLINs on pregnancy outcomes, further research with larger sample sizes, may be needed to add to the evidence base for finalizing WHO’s recommendation on PBO LLINs.

## Data Availability

The datasets used and/or analyzed during the current study are available from the corresponding author on reasonable request.

## Abbreviations

CYP450: Cytochrome P450
HIV: Human immunodeficiency virus
HMIS: Health Management Information System
HSD: Health subdistrict
IRR: Incidence rate ratio
ITSA: Interrupted time series analyses
kdr: Knockdown resistance
LBW: Low birthweight
LLIN: Long-lasting insecticidal net
PBO: Piperonyl butoxide
WHO: World Health Organization
